# Trichloroethylene Intoxication: A Case of Acute Altered Mental Status Requiring Intensive Resuscitation

**DOI:** 10.7759/cureus.77215

**Published:** 2025-01-10

**Authors:** Atsuhito Tanaka, Ji Young Huh, Koichi Ariyoshi

**Affiliations:** 1 Department of Emergency and Critical Care Medicine, Tokyo Bay Urayasu Ichikawa Medical Center, Chiba, JPN; 2 Emergency Department, Kobe City Medical Center General Hospital, Kobe, JPN

**Keywords:** hydrocarbon poisoning, medical intensive care unit (micu), toxicology and poisoning, trichloroethylene, work-related injury

## Abstract

Trichloroethylene (TCE) is a hydrocarbon used commercially as a degreasing solvent. It has gradually become obsolete due to its carcinogenic properties, but many industries still use it given its cost-effectiveness. While there have been reports and studies regarding chronic exposures to TCE, very few acute cases of altered mental status requiring intensive care have been reported so far. We discuss the course of an acute case of TCE intoxication with coma and summarize its characteristics.

A 50-year-old male with no previous medical history presented with acute altered mental status due to inhalation of volatile TCE in a factory. He was given brief cardiopulmonary resuscitation for suspected cardiac arrest; however, he was found to have a pulse when emergency medical services intervened. On arrival at our emergency department, he showed marked central nervous system (CNS) depression, raising concerns for acute TCE intoxication. The patient was intubated promptly, given the expected impending respiratory arrest, and received supportive treatment. TCE metabolite urinary concentrations were taken during admission. He regained consciousness readily, was extubated the next day, and then discharged after showing full recovery on day three.

This patient with acute TCE intoxication required intensive care but was discharged with full recovery and no neurological deficits. There have been few reports describing acute intoxication needing intubation and its neurological outcomes. Furthermore, this report suggests urine metabolite concentration as a potential marker to predict clinical outcomes in these patients.

## Introduction

Trichloroethylene (TCE) is a clear, colorless nonflammable liquid that was historically used as an anesthetic and later as a degreasing solvent commercially. However, its carcinogenic properties and associated health issues became evident in the 1990s, largely reducing the use of this solvent in industries. Nevertheless, some companies still use TCE due to its cost-effectiveness [1]. To date, very few cases of acute TCE intoxication have been reported clinically. We present a case of a patient who underwent intubation and supportive treatment due to central nervous system (CNS) depression secondary to inhalation of volatile TCE. We discuss the clinical course and successful resuscitation experience.

This article was previously posted to the Research Square preprint server on July 21, 2022.

## Case presentation

**Figure 1:**
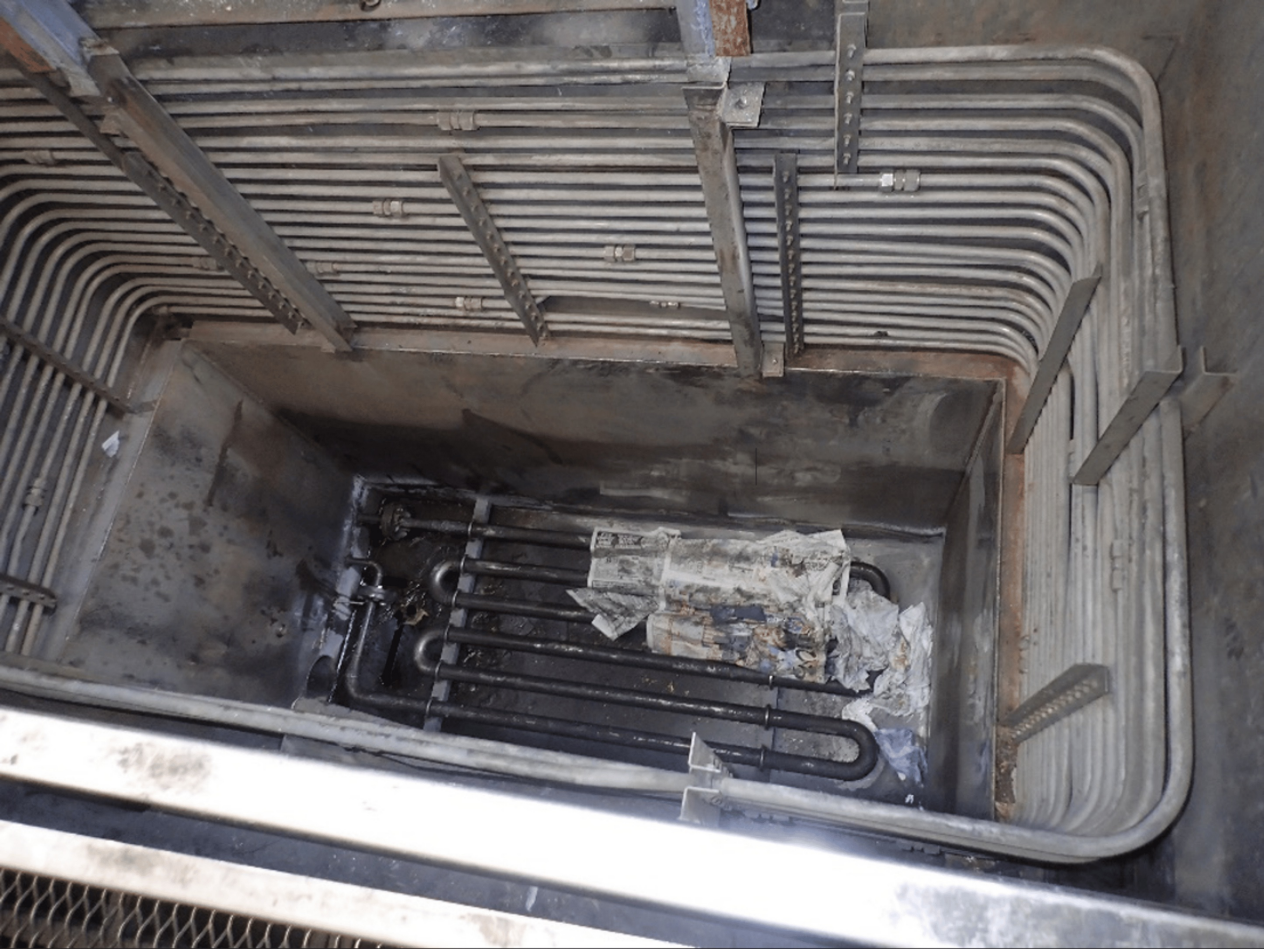
Original container

A 50-year-old male was brought into our hospital due to sudden altered mental status. The patient worked in a factory that uses TCE and keeps the waste product in a large concrete container outside. The original container used is shown in Figure 1.

The container is maintained by scraping the residue TCE from the floor while ventilating the area. However, the patient had failed to take adequate safety measures and was found unconscious on the container floor by his coworkers and received brief cardiopulmonary resuscitation. The emergency medical service on arrival judged the patient to have a pulse and he was triaged to our hospital.

On arrival, the patient’s initial vital signs were as follows - Glasgow Coma Scale score: E1V1M1, blood pressure: 103/74 mmHg, heart rate: 80 beats per minute sinus rhythm, respiratory rate: 10 times per minute, and oxygen saturation: 95% on 15L per minute of oxygen. The arterial blood gas indicated mixed acidosis (pH: 7.253, partial pressure of carbon dioxide: 50.0 mmHg, partial pressure of oxygen: 305.0 mmHg, and bicarbonate: 19.8 mEq/L). It showed no traces of carbon monoxide. Blood tests revealed leukocytosis (white blood cell count: 18,700 /μL), elevated liver function (aspartate aminotransferase: 126 IU/L, alanine aminotransferase: 95 IU/L), and renal failure (creatinine: 1.44 mg/dL, estimated glomerular filtration rate: 43 mL/min/1.73m^2^). ECG showed normal sinus rhythm with no arrhythmias and echocardiography showed normal ejection fraction with no obvious asynergy or dyskinesia. High-sensitive troponin was within normal limits. Urine toxic screening was negative.

Intubation was indicated due to CNS depression, protection of the airway, and decreased respiratory rate. Head and abdominal CT scans showed no abnormalities. Chest CT showed mild aspiration patterns. The patient was admitted to the ICU for the treatment of TCE intoxication. On ICU day one, the patient regained full consciousness under intubation and was extubated successfully. His blood tests including mixed acidosis, liver enzymes, and renal function all greatly improved (Table 1).

**Table 1:** Blood test results ER: emergency room; WBC: white blood cell; Hb: hemoglobin; Plt: platelet; Na: sodium; K: potassium; CRP: C-reactive protein; AST: aspartate aminotransferase; ALT: alanine aminotransferase; CK: creatinine kinase; Cr: creatinine; eGFR: estimated glomerular filtration rate; BUN: blood urea nitrogen; Amy: amylase

Variable	ER	Day 1	Day 2	Day 3
WBC (10^3^/μL)	18.7	14.1	8.6	5.6
Hb (g/dL)	14.9	13.3	11.5	12.7
Plt (10^4^/μL)	17.1	22.6	16.1	19.1
Na (mEq/L)	139	139	142	144
K (mEq/L)	4.6	4.5	3.3	3.7
CRP (mg/dL)	0.04	1.4	1.67	0.69
AST (U/L)	126	100	51	45
ALT (U/L)	95	79	50	47
CK (U/L)	272	1172	513	358
CKMB (U/L)	62.5	21		
Cr (mg/dL)	1.44	0.98	0.86	0.94
eGFR (mL/min/1.73^2^)	43	65	75	68
BUN (mg/dL)	14.8	12.5	14.1	11.5
Amy (U/L)	510	161	126	135

**Figure 2:**
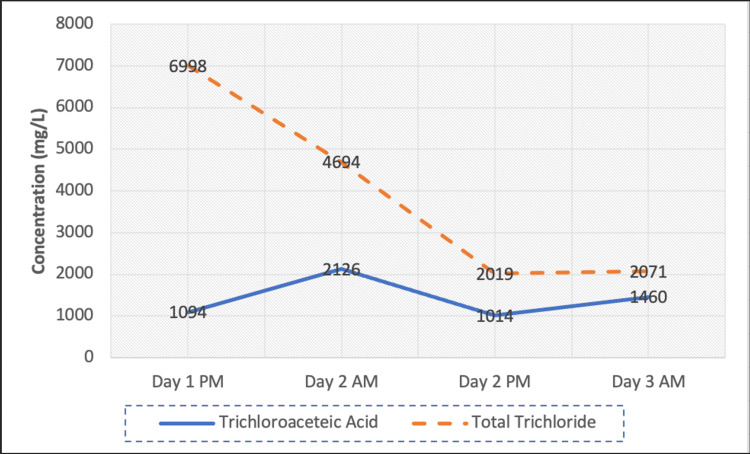
Concentration of trichloroacetic acid and total trichloride

The patient was given a mental state exam on day three; he scored 28/30 on the Montreal Cognitive Assessment (MoCA), indicating minimal damage to neurological function. The patient recovered without any obvious complications and was discharged. Urinary trichloroacetic acid (TCA) and total trichloride (TTC) concentrations were also measured at 12-hour intervals and showed steady excretion; however, he was discharged before the total trend could be observed (Figure 2). He was eventually lost to outpatient follow-up.

## Discussion

We report a case of acute TCE intoxication with severe altered mental status requiring intensive care. In TCE poisoning, inhalation exposure is the most important entry route and its toxicity mainly affects the nervous system, particularly depressing CNS functions. TCE acts on the CNS by inhibiting receptor function at nicotinic and glutamate receptors as well as enhancing type A γ-aminobutyric acid and glycine receptor currents, thus causing CNS depression [2]. At high doses, TCE exhibits anesthetizing properties, resulting in respiratory depression and death in some cases. It is metabolized by the liver into TTC and TTE, which are excreted into urine [3]. Reversible neurological dysfunction after acute exposure in mice has been confirmed, while in cases of heavy exposure, respiratory arrest has been noted [4]. Our patient was found in a TCE vapor-filled container where the concentration of TCE was unknown. After admission, his level of consciousness gradually improved accordingly with a decrease in metabolite concentration. Thus, we can infer that the main reason for altered mental status in this case was CNS depression from TCE exposure.

However, the possibility of cardiotoxic effects causing an arrest and subsequent loss of consciousness cannot be fully ruled out. Several case reports have documented death due to TCE inhalation. These fatalities are thought to have been caused by cardiac arrhythmias, due to a lack of demonstrable autopsy findings and animal studies of arrhythmias presenting before death [5]. The underlying mechanism of cardiac arrhythmias is thought to be TCE blocking the potassium current, prolonging repolarization, sensitizing the myocardium to endogenous and administered catecholamines with the potential for life-threatening ventricular arrhythmia [6]. In this case, however, the initial cardiac rhythm showed sinus waves, and no cardiac events were observed throughout admission. Head CT also showed no signs of hypoxic-ischemic encephalopathy. Given that 80% of patients admitted to an ICU after resuscitation from out-of-hospital cardiac arrest are comatose and two-thirds die due to hypoxic-ischemic brain injury, the possibility of the patient going into cardiac arrest is unlikely [7].

There are mixed conclusions regarding the chronic neurologic effects of TCE exposure. Research on humans describes chronic exposure causing mild neurological changes (decrease in perception, dexterity, reaction times) that were reversible while mice showed permanent cognitive damage [6,8]. On the other hand, a few reports of acute exposure have described neurological changes, especially in severe cases where intubation was suggested due to CNS depression. In this case, the patient showed acute altered mental status but readily recovered several hours after initial resuscitation and intubation. His mental state and neurological function also showed no marked damage. Although a follow-up examination is warranted to verify long-term effects, acute intoxication may not cause irreversible neurological damage even in severe exposures.

Very few clinical cases of acute TCE exposure and urine concentration of its metabolites have been reported. Previous studies regarding TCE hemodynamics in humans have described the association between the urinary concentration of TCE and its metabolites as linear but largely affected by the individual. Literature suggests that the terminal half-life of TCE is 30-38 hours; however, its metabolites may persist in urine for up to several weeks [9,10]. In this particular case, the patient showed quick recovery in all systems within several days of admission. Urinary concentration of TTC suggests a proportional relationship between clinical symptoms; however, TCE concentration remained at a stable level throughout admission. This may be due to the proportion of metabolites of TCE; TTC only accounts for roughly 8%, whilst TTC accounts for over 90% [11]. Further urinary concentrations of TCE metabolites need to be traced for several weeks to track the clinical relationship.

## Conclusions

Very few acute cases of TCE intoxication needing intensive care have been reported so far. This report provides crucial insights into the clinical course and treatment of TCE intoxication. Even though the reason for altered mental status is unclear, even in cases of acute TCE exposure which causes CNS depression, irreversible damage may be avoided. Furthermore, urine metabolite concentrations could serve as a marker to predict clinical symptom recovery.
